# The Cut-Off Values for SHBG Discriminating Insulin Resistance Based on the TyG, TyG-BMI, and TyG-WC Values in Women with PCOS

**DOI:** 10.3390/biomedicines14010187

**Published:** 2026-01-15

**Authors:** Marta Kochanowicz, Tahar Ben Rhaiem, Aleksander J. Owczarek, Mariusz Wójtowicz, Paweł Madej, Jerzy T. Chudek, Magdalena Olszanecka-Glinianowicz

**Affiliations:** 1 Department of Gynecological Endocrinology, Faculty of Medical Sciences in Katowice, Medical University of Silesia in Katowice, 40-752 Katowice, Poland; 2 Health Promotion and Obesity Management Unit, Department of Pathophysiology, Faculty of Medical Sciences in Katowice, Medical University of Silesia, 40-752 Katowice, Poland; 3 Clinical Department of Gynecology and Obstetrics, Faculty of Medical Sciences in Zabrze, Medical University of Silesia, Katowice, 41-803 Zabrze, Poland; 4 Department of Internal Medicine and Oncological Chemotherapy, Faculty of Medical Sciences in Katowice, Medical University of Silesia, 40-029 Katowice, Poland

**Keywords:** insulin resistance, TyG, TyG-BMI, TyG-WC, SHBG, PCOS

## Abstract

**Background**: Recently, based on HOMA-IR, we estimated empirical optimal cut-off values for SHBG levels of ≤41.5 nmol/L in women with PCOS. Other proposed markers of insulin resistance include triglyceride and glucose levels, and anthropometric measurements. Therefore, our current study aimed to analyze its consistency with the cut-off values that discriminate insulin resistance based on the TyG, TyG-BMI, and TyG-WC indices in women with PCOS. **Methods:** Age, body weight, height, waist circumference, glucose, insulin, triglyceride, and SHBG levels were retrieved from the medical records of 264 Caucasian women diagnosed with PCOS. The TyG, TyG-BMI, and TyG-WC indices were calculated. The mean meta-cut-off SHBG level was calculated using receiver-operating characteristic (ROC) analysis combined with diagnostic test accuracy meta-analysis. **Results**: The mean meta-cut-off value for SHBG levels for the assessment of insulin resistance was less than 43.1 (95% CI: 37.0–49.2) nmol/L. The pooled sensitivity and specificity of SHBG levels for the assessment of insulin resistance were 74.7% and 66.9%, respectively. The pooled mean prevalence of insulin resistance based on all indices was 36.1% (95% CI: 33.5–38.7%) with a standard deviation of 18.7% and positive predictive value (PPV) of 52.8% (95% CI: 12.2–87.5%) and the negative predictive value (NPV) of 80.2% (95% CI: 45.1–97.7%). **Conclusions**: Our study confirms the usefulness of SHBG level as a marker of insulin resistance in Caucasian women with PCOS. A value below 43 nmol/L, with high sensitivity and specificity, enables the detection of insulin resistance and a high risk of prediabetes, prompting close monitoring of liver function.

## 1. Introduction

Hepatokines are a group of secreted proteins related to glucose and lipid metabolism, synthesized by hepatocytes, including fetuin A, fibroblast growth factor-21 (FGF-21), selenoprotein P, sex hormone-binding globulin (SHBG), angiopoietin-related growth factor (AGF), leukocyte cell-derived chemotaxin-2 (LECT2), follistatin, heat shock protein (HSP76), inhibin betaE, insulin-like growth factor type 1 (IGF-1), IGF-1 binding protein (IGFBP-1), angiopoietin-like factor type 4 (ANGPTL-4) [1].

SHBG is a homodimeric glycoprotein belonging to the fraction of plasma beta-globulins with high affinity for androgens and estrogens produced mainly by the liver [2]. Androgens, insulin, IGF-1, and prolactin inhibit SHBG synthesis, while estrogens and thyroxine stimulate it [3,4,5,6]. The circulating concentration of SHBG depends on gender, age, and the laboratory method used for its determination [5,6].

Hyperinsulinemia, compensating for insulin resistance developing as a result of excessive visceral fat deposition, inhibits the synthesis of SHBG in the liver, which is why low concentrations of this protein are considered a risk factor for the development of type 2 diabetes [7,8,9,10,11,12]. On the other hand, SHBG can be regarded as a marker of hepatic insulin resistance, because its concentration is inversely proportional to the degree of fatty liver, insulin concentrations, and HOMA-IR values [13,14,15,16,17]. It should also be mentioned that experimental studies have shown that SHBG regulates the activity of glucose transporters and, in this mechanism, may regulate its metabolism [18]. Recently, based on HOMA-IR, we estimated empirical optimal cut-off values for SHBG levels of ≤41.5 nmol/L in women with PCOS [19]. As hepatic steatosis results in insulin resistance, it is associated with impaired utilization of fatty acids, increased serum triglyceride levels, and intensified gluconeogenesis with impaired glucose storage as glycogen. The triglyceride–glucose index (TyG) was proposed as a marker of liver steatosis and insulin resistance [20]. Later, an analysis of data from the Korean National Health and Nutrition Examination Survey, including 11,149 adults, showed that multiplying the TyG value by parameters such as BMI and waist circumference may be used as markers of hepatic insulin resistance. In addition, the TyG-BMI index turned out to be a better marker of insulin resistance than TyG [21]. While the analysis of data from the National Health and Nutrition Examination Survey (NHANES) conducted in 2017–2018, including 1727 adults, confirmed its usefulness in early screening for NAFLD and MAFLD and in monitoring disease progression [22]. Therefore, our current study aimed to analyse its consistency with the cut-off values that discriminate insulin resistance based on the TyG, TyG-BMI, and TyG-WC indices in women with PCOS.

## 2. Materials and Methods

The retrospective study includes data from the medical records of 311 consecutive Caucasian women for the first time diagnosed with PCOS based on the Rotterdam criteria [23] and hospitalized at the Department of Gynecological Endocrinology in 2019–2021. This study did not require patient consent according to the Polish law, and inclusion of all hospitalized patients with the final diagnosis in a specific time period that precluded selection bias.

Women with phenotype A constituted 58.6%, phenotype B 11.8%, phenotype C 15.3%, and phenotype D 14.3% of the study group.

The inclusion criteria comprised individuals aged 18–40 years and a diagnosis of PCOS. The exclusion criteria were diagnosis of arterial hypertension, type 2 diabetes, and other endocrinological disturbances (including hypothyroidism, congenital adrenal hyperplasia, hyperprolactinemia, and POI), any pharmacological therapy, treatment of obesity in the past and currently, and the lack of necessary data in the medical records.

The analyzed data set included age, body mass, height, waist circumference, and routine measurements of fasting glucose, triglycerides, insulin, and SHBG levels, all performed in a single hospital laboratory using the same set of methods for all study subjects. Glucose and triglyceride concentrations were measured using the colorimetric method (Roche reagents). Insulin and SHBG levels were determined using the ECLIA method (Roche Diagnostics GmbH, Mannheim, Germany, reagents for Cobas E411). BMI, HOMA-IR, TyG, TyG-BMI, and TyG-WC indices values were calculated with standard formulas:

HOMA-IR = fasting insulin level [uIU/mL] × fasting glucose level [mg/dL]/405

TyG = Ln[fasting triglycerides (mg/dL) × fasting glucose (mg/dL)/2];

TyG-BMI = Ln[fasting triglycerides (mg/dL) × fasting glucose (mg/dL)/2] × BMI,

TyG-WC = Ln[fasting triglycerides (mg/dL) × fasting glucose (mg/dL)/2] × WC.

As the retrospective analysis of patients’ records in accordance with the regulations of the Medical University of Silesia in Katowice does not meet the criteria of a medical experiment, the approval of the Bioethical Committee and patients’ consent were not required.

### 2.1. Data Analysis

During the analysis of medical records of 311 women with PCOS, it was found that 35 (11.2%) were diagnosed with thyroid diseases, 2 (0.6%) with type 1 diabetes, 5 (1.6%) with type 2 diabetes, and 8 (2.6%) with hypertension. According to the exclusion criteria, they were excluded from further analysis. Finally, data from 264 women were analyzed. The cut-off points for insulin resistance were a TyG index of >8.55 for the general population [24] and >8.51 for women with PCOS [25]. The second marker of insulin resistance was a TyG-BMI index of >237.77, estimated for the general population [22], and >191.53, estimated for women with PCOS [25]. The third marker of insulin resistance was the TyG-WC index > 822.34 estimated for the general population [22].

### 2.2. Statistical Analysis

Statistical analysis was performed using STATISTICA 13.0 PL (TIBCO Software Inc., Palo Alto, CA, USA), StataSE 13.0 (StataCorp LP, College Station, TX, USA), and R software v. 4.5.1 (R Core Team 2013, R: A language and environment for statistical computing. R Foundation for Statistical Computing, Vienna, Austria. URL http://www.R-project.org/, accessed on 13 January 2026). Statistical significance was set at a *p*-value below 0.05. All tests were two-tailed. Imputations were not done for missing data. The meta-analysis of diagnostic test accuracy and sensitivity analysis was done with DTAmetsa 1.0.0 software [26] and Meta-Disc 2.0 software [27]. Wilson score interval method of variance calculation for the binomial proportion confidence interval and Deek’s method detecting bias on the ln(DOR) were used. Interval data were compared between independent groups with the Student *t*-test for data with normal distribution or with the Mann–Whitney U test otherwise. Data normality was assessed with the Shapiro–Wilk test and quantile-quantile plot. Nominal or ordinal data were compared with the χ^2^ test. The summary ROC curve (sROC curve) for the bivariate model with data points (sensitivity and specificity for each index), summary estimate, 95% confidence region (CI), and 95% prediction region were used to present results from diagnostic test accuracy meta-analysis.

## 3. Results

Study group characteristics are shown in Table 1. The cut-offs for serum SHBG levels estimated based on different, adopted from recent publication cut-offs for TyG indices and HOMA-IR, are presented in Table 2.

**Figure 1:**
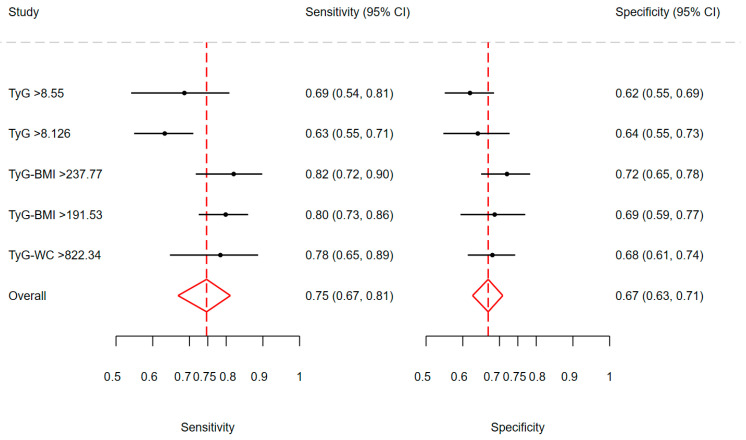
The univariate forest plots for sensitivity and specificity values for each index with confidence intervals (black lines). The red dashed line shows the weighted mean of sensitivity and specificity, and the red triangles show the overall weighted sensitivity and specificity (width is a confidence interval), respectively.

The highest AUC was obtained for TyG-BMI (≥237.77), the lowest for TyG (≥8.126). The highest sensitivity and specificity were also noted for TyG-BMI (≥237.77), while the lowest sensitivity was for TyG (≥8.126) and the lowest specificity for TyG (≥8.55). There was a significant difference in sensitivity values yielded by each index (χ^2^ = 15.27; df = 4; *p* < 0.01), but not in specificity values (χ^2^ = 5.28; df = 4; *p* = 0.26). The correlation between sensitivity and specificity on the logit scale was 1. In addition, we also present cut-off for SHBG for HOMA-IR ≥ 2.1 (Table 1). Figure 1 presents the univariate forest plots for sensitivity and specificity values for each TyG-based index.

### Diagnostic Test Accuracy

A diagnostic test accuracy meta-analysis of diagnostic test accuracy based on the mentioned indices was conducted using both univariate and bivariate models. In univariate analysis, the sensitivity and specificity were 74.8% (95% CI: 67.3–81.0%) and 67.0% (95% CI: 63.5–70.3%), respectively. The received sensitivity and specificity are comparable with the corresponding values obtained for HOMA-IR. The diagnostic odds ratio was 6.041 (95% CI: 3.536–10.322), with Cochran’s Q value of 3.748 (df = 4; *p* = 0.44) and Higgins’ I^2^ = 0%, meaning the low heterogeneity of DORs between indices. Overall, patients with SHBG levels below cut-offs for TyG-based indices had more than six times higher odds of being insulin resistant. However, there was more heterogeneity in sensitivity (χ^2^ = 0.104, I^2^ = 73.1%) than in specificity (χ^2^ = 0.004, I^2^ = 23.8%). A summary of the bivariate analysis is presented in Table 3. Coefficients and the heterogeneity measures are summarized in Table 4 and Table 5.

The pooled sensitivity and specificity of SHBG levels for the assessment of insulin resistance were 74.7% and 66.9%, respectively. The pooled mean prevalence of insulin resistance based on each index was 36.1% (95% CI: 33.5–38.7%) with a standard deviation of 18.7%. Based on this assessment, the pooled positive predictive value (PPV) was 52.8% (95% CI: 12.2–87.5%), that is lower than the value obtained for HOMA-IR (79.0%); and the negative predictive value (NPV) was 80.2% (95% CI: 45.1–97.7%), which is higher than for HOMA-IR (64.3%).

**Figure 2:**
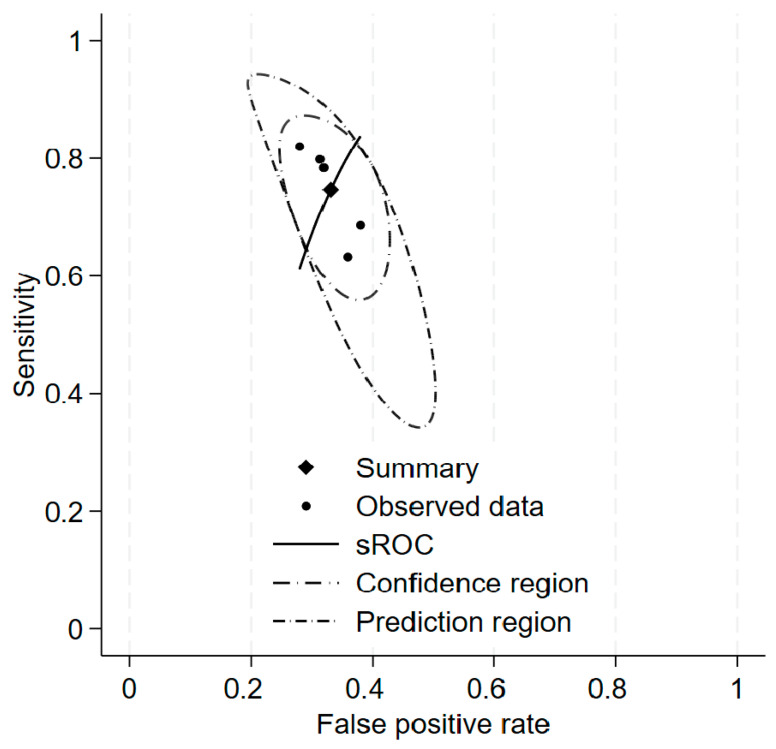
The sROC curve for the bivariate model with data points (sensitivity and specificity for each index), summary estimate, 95% confidence region, and 95% prediction region.

In the bivariate analysis, there was also more heterogeneity in sensitivity (χ^2^ = 0.120, I^2^ = 62.6%) than in specificity (χ^2^ = 0.018, I^2^ = 38.3%). So, the ability of TyG-dependent indices to correctly identify subjects without insulin resistance is more consistent between indices. Despite the presence of heterogeneity in both dimensions, the generalized bivariate heterogeneity I^2^ was only 1%. This is due to the perfect correlation between sensitivity and specificity, and because the generalized between-study variance goes to 0. The summary ROC (sROC) curve is presented in Figure 2.

The AUC for this curve was 0.733, and the partial AUC (restricted to observed FPRs and normalized) was 0.729. The mean meta-cut-off value for SHBG levels for the assessment of insulin resistance was less than 43.1 (95% CI: 37.0–49.2) [nmol/L].

After stratification into subgroups according to the established cut-off point of SHBG level for insulin resistance < 43.1 nmol/L, we found that this subgroup consisted of 48.1% of the analyzed cohort. This subgroup was characterized by significantly higher body mass, BMI, and waist circumference, as well as frequent diagnosis of overweight and obesity based on BMI values and visceral obesity based on waist circumference. There were no differences in glucose levels and frequency of impaired fasting glucose between subgroups with SHBG levels < 43.1 nmol/L and > 43.1 nmol/L. Significantly higher serum insulin and triglyceride levels, as well as frequent HOMA-IR > 2.1 and hypertriglyceridemia, were found in the subgroup with SHBG levels < 43.1 nmol/L (Table 6).

## 4. Discussion

To the best of our knowledge, this is the first diagnostic test accuracy meta-analysis study estimating the cut-off value for SHBG levels discriminating insulin resistance based on various indices (TyG, TyG-BMI, and TyG-WC) in women with PCOS. Previously, this point has been estimated based on the HOMA-IR value. Recently, we demonstrated that an SHBG level of 41.5 mmol/L or less, despite its low sensitivity, is quite specific, and therefore can be considered a marker of insulin resistance in women with PCOS [19]. In the present study, the mean meta-cut-off value for SHBG levels for the assessment of insulin resistance was less than 43.1 (95% CI: 37.0–49.2) nmol/L. It should be noted that in this analysis, the TyG and TyG-BMI cut-off points we used for the assessment of insulin resistance derived from the general population and women with PCOS, and TyG-WC from the general population only. Of note, the cut-off points for SHBG, calculated in this study, were similar to those previously determined based on the HOMA-IR value [19] but significantly higher than the widely accepted lower limit of the laboratory norm (26.1 nmol/L).

The pooled sensitivity and specificity of SHBG levels for the assessment of insulin resistance were 74.7% and 66.9%, respectively. Based on this analysis, the pooled positive predictive value (PPV) was 52.8% (95% CI: 12.2–87.5%), and the negative predictive value (NPV) was 80.2% (95% CI: 45.1–97.7%). This suggests that SHBG levels are more effective at excluding insulin resistance than diagnosing it. It should be noted that the pooled sensitivity for the mean cut-off points for SHBG levels determining insulin resistance was higher than the sensitivity for the cut-off point for SHBG levels estimated based on HOMA-IR value (61.1%). While the specificity was lower (71.6%) [19].

The SHBG cut-off point below 43 nmol/L estimated empirically in our study is located between those estimated in women with PCOS for assessment of the risk of the development of NAFLD (below 30 nmol/L) [16] and the risk of type 2 diabetes development in the general population (below 50 nmol/L) [12].

Our study confirmed the usefulness of SHBG level, being a part of the PCOS diagnostic panel, as a marker of insulin resistance, especially considering the much lower variability of SHBG compared to glucose and insulin concentrations [28]. Moreover, Borai et al. [29] found that we may be too hasty in concluding insulin resistance based on HOMA-IR values because it depends on the laboratory method of measuring insulin levels. It was previously shown that the distribution of HOMA-IR values differed by up to twofold, depending on the method of measuring insulin concentration [30]. Moreover, the comparison of 11 laboratory methods for measuring insulin levels revealed that this discrepancy may be due to variable specificity, different calibration settings, and even varying formulas used to convert insulin units [31].

The main limitation of the present study is the lack of imaging assessment of fatty liver, which is a cause of liver insulin resistance development and decreased SHBG synthesis. Moreover, we used the TyG, TyG-BMI, and TyG-WC cut-off points estimated for the general population, as well as the TyG and TyG-BMI cut-off points estimated in Asian women with PCOS, for assessing insulin resistance in Caucasian women, due to the lack of estimates for this population. On the other hand, a strength of our study is the large size of the study group and the inclusion of a homogeneous group of young Caucasian women diagnosed with PCOS, with different nutritional statuses.

## 5. Conclusions

Our study confirms the usefulness of SHBG level as a marker of insulin resistance in Caucasian women with PCOS. A value below 43 nmol/L, with high sensitivity and specificity, enables the detection of insulin resistance and a high risk of prediabetes, prompting close monitoring of liver function.

## Figures and Tables

**Table 1:** Characteristics of the study group [N = 264].

Parameter	Value
Age [years]	26 + 5
Phenotypes of PCOS [N/(%)]	
A	155 (58.6)
B	31 (11.8)
C	40 (15.3)
D	38 (14.3)
Hirsutism based on Ferriman and Gallwey scale > 8 points [N/(%)]	123 (46.6)
LH/FSH	1.12 [0.85–1.54]
Testosterone [ng/mL]	0.347 [0.264–0.476]
FAI	0.771 [0.454–1.411]
BMI [kg/m^2^]	24.8 [21.3–29.4]
Overweight [N/(%)]	68 (25.8)
Obesity [N/(%)]	62 (23.4)
Waist circumference [cm]	84.9 + 15.9
Visceral obesity [N/(%)]	144 (54.6)
Triglycerides [mg/dL]	95.3 + 49.0
Glucose [mg/dL]	84.9 ± 5.8
Insulin [uIU/mL]	7.9 [5.6–12.0]
HOMA-IR	2.2 + 2.0
HOMA-IR ≥ 2.1 [N/(%)]	98 (37.1)
SHBG [nmol/L]	46.4 [30.2–64.8]
TyG	8.19 + 0.45
TyG-BMI	217.9 [171.2–248.2]
TyG-WC	667 [576–787]

Mean ± standard deviation or median [lower quartile–upper quartile].

**Table 2:** Diagnostic performance (accuracy, sensitivity, and specificity) of the cut-offs for SHBG levels for insulin resistance assessment based on cut-offs for TyG indices and HOMA-IR.

	TyG ≥22658.55	TyG ≥8.126	TyG × BMI ≥237.77	TyG × BMI ≥191.53	TyG × WC ≥822.34	HOMA-IR ≥2.1
N of 264 (%)	51 (19.3)	147 (55.7)	78 (29.5)	149 (56.4)	51 (19.3)	98 (37.1)
Area under curve (AUC) [%]	73.6 (66.0–81.2)	67.5 (61.1–74.0)	84.5 (79.7–89.4)	82.8 (77.8–87.7)	83.2 (77.6–88.8)	78.9 (73.3–84.5)
Cut-off for SHBG	<40.24	<45.23	<40.12	<50.81	<39.05	<36.8
level [nmol/L]						
Youden index	0.306	0.274	0.541	0.486	0.465	0.438
Prevalence [%]	19.3 (15.0–24.5)	55.7 (49.7–61.6)	29.6 (24.4–35.3)	56.4 (50.4–62.3)	19.3 (15.0 –24.5)	63.1 (57.1–69.0)
Accuracy [%]	63.3	63.6	75.0	75.0	70.1	73.6
True positive [N]	35	93	64	119	40	132
False negative [N]	16	54	14	30	11	35
False positive [N]	81	42	52	36	68	35
True negative [N]	132	75	134	79	145	63
Sensitivity [%]	68.6 (55.0–79.7)	63.3 (55.2–70.6)	82.1 (72.1–89.0)	79.9 (72.7–85.5)	78.4 (65.4–87.5)	79.0 (71.9–84.8)
Specificity [%]	62.0 (55.3–68.2)	64.1 (55.1–72.2)	72.0 (65.2–78.0)	68.7 (59.7–76.5)	68.1 (61.5 –74.0)	64.3 (53.9–73.5)
False positive rate [%]	38.0 (31.8–44.7)	35.9 (27.8–44.9)	27.9 (22.0–34.8)	31.3 (23.5–40.3)	31.9 (26.0 –38.4)	37.9 (31.2–43.1)
Positive predictive value [%]	30.1 (22.2–39.5)	68.9 (60.3–76.4)	55.2 (45.7–64.3)	76.8 (69.2–83.0)	37.0 (28.1 –46.9)	79.0 (71.9–84.8)
Negative predictive value [%]	89.2 (92.8–93.5)	58.1 (49.1–66.7)	90.5 (84.3–94.5)	72.5 (62.9–80.4)	92.9 (87.4 –96.2)	64.3 (53.9–73.5)
DOR (≥cut-off)	3.56 (1.86–6.85)	3.07 (1.86–5.10)	11.78 (6.08–22.82)	8.70 (4.96–15.27)	7.75 (3.75 -16.04)	6.99 (4.00–12.22)
*p*-value for DOR	<0.001	<0.001	<0.001	<0.001	<0.001	<0.001

Values are presented with 95% confidence intervals: AUC—Area under curve, DOR—diagnostic odds ratio, SHBG—sex hormone-binding globulin, Youden index—composite performance measure [=sensitivity + specificity − 1].

**Table 3:** Bivariate analysis—summary statistics of diagnostic test accuracy.

	Estimate	95% LCI	95% UCI
Sensitivity [%]	74.7	66.9	81.1
Specificity [%]	66.9	62.7	70.9
DOR	5.962	3.628	9.798
LR+	2.257	1.865	2.732
LR−	0.379	0.276	0.52
FPR	0.331	0.291	0.373

DOR—diagnostic odds ratio, LR—likelihood ratio, FPR—false positive ratio, LCI/UCI—lower/upper confidence interval.

**Table 4:** Bivariate analysis—summary statistics.

	Estimate
Logit sensitivity	1.081
Logit specificity	0.705
Var (logit [sensitivity])	0.120
Var (logit [specificity])	0.018
SE (logit [sensitivity])	0.193
SE (logit [specificity])	0.095
Correlation	1

Var—variance, SE—standard error.

**Table 5:** Bivariate analysis—heterogeneity.

	Estimate
Var logit(sensitivity)	0.120
Var logit(specificity)	0.018
MOR sensitivity	1.392
MOR specificity	1.137
Bivariate I^2^	0.010
Area 95% Prediction Ellipse	0.082

MOR—median odds ratio.

**Table 6:** Characteristics of the study group with and without insulin resistance based on the meta cut-off point of SHBG level.

	SHBG < 43.1	SHBG ≥ 43.1	*p*
N (%)	127 (48.1)	137 (51.9)	
Age [years]	25.2 ± 4.6	26.2 ± 4.8	0.08
Body weight [kg]	83.3 ± 21.2	64.0 ± 12.8	<0.001
BMI [kg/m^2^]	29.8 ± 7.0	23.3 ± 2.6	<0.001
Normal weight [N/(%)]	34 (26.8)	100 (73.0)	
Overweight [N/(%)]	41 (32.3)	27 (19.7)	<0.001
Obesity [N/(%)]	52 (40.9)	10 (7.3)	
Waist circumference [cm]	92.4 ± 17.2	77.6 ± 10.9	<0.001
Visceral obesity [N/(%)]	96 (75.6)	48 (35.0)	<0.001
Glucose [mg/dL]	85.0 ± 5.7	84.8 ± 6.0	0.75
Impaired fasting glucose [N/(%)]	2 (1.6)	2 (1.5)	1.00
Insulin [uIU/mL]	10.4 (7.5; 14.8)	6.4 (4.7; 8.4)	<0.001
HOMA-IR	2.26 (1.60; 3.15)	1.33 (1.00; 1.73)	<0.001
HOMA-IR ≥ 2.1 [N/(%)]	72 (56.7)	26 (19.0)	<0.001
Triglycerides [mg/dL]	97.7 (69.4; 130.0)	75.4 (57.1; 96.8)	<0.001
Hypertriglyceridemia [N/(%)]	19 (15.0)	6 (4.4)	<0.01

BMI—body mass index, HOMA-IR—homeostatic model assessment—insulin resistance, SHBG—sex hormone-binding globulin.

## Data Availability

The raw data supporting the conclusions of this article will be made available by the authors on request.

## Abbreviations

The following abbreviations are used in this manuscript:

AGF	Angiopoietin-related growth factor
ANGPTL-4	Angiopoietin-like factor type 4
BMI	Body mass index
CI	Coefficient interval
DOR	Diagnostic odds ratio
FGF-21	Fibroblast growth factor-21
FPR	False positive ratio
HOMA-IR	Homeostatic Model Assessment–Insulin Resistance
HSP76	Heat shock protein
IGFBP-1	IGF-1 binding protein-1
IGF-1	Insulin-like growth factor type 1
LCI/UCI	Lower/upper confidence interval
LECT-2	Leukocyte cell-derived chemotaxin-2
LR	Likelihood ratio
MOR	Median odds ratio
NPV	Negative predictive value
PCOS	Polycystic ovary syndrome
PPV	Positive predictive value
ROC	Receiver-operating characteristics
SE	Standard error
SHBG	Sex hormone-binding globulin
TyG	Triglyceride glucose index
TyG-BMI	TyG-body mass index
TyG-WC	TyG-waist circumference index
Var	Variance
